# Endophytic Fungal Community of *Huperzia serrata*: Diversity and Relevance to the Production of Huperzine A by the Plant Host

**DOI:** 10.3390/molecules26040892

**Published:** 2021-02-08

**Authors:** Lingli Cui, Hamza Armghan Noushahi, Yipeng Zhang, Jinxin Liu, Andreea Cosoveanu, Ying Liu, Ling Yan, Jing Zhang, Shaohua Shu

**Affiliations:** 1 College of Plant Science & Technology, Huazhong Agricultural University, Wuhan 430070, China; cuilingli2021@163.com (L.C.); hamzanaushahi143@gmail.com (H.A.N.); zypzxcvb67890@163.com (Y.Z.); mrjinxinliu@outlook.com (J.L.); 18186421026@163.com (Y.L.); Lingy0103@gmail.com (L.Y.); zhangjingupup@163.com (J.Z.); 2 Laboratory of Useful Organisms, Research—Development Institute for Plant Protection, Ion Ionescu de la Brad no. 8 Blvd., 013813 Bucharest, Romania; acosovea@ull.edu.es

**Keywords:** huperzine A, *Huperzia serrata*, endophytic fungi, diversity, Alzheimer’s disease

## Abstract

As the population ages globally, there seem to be more people with Alzheimer’s disease. Unfortunately, there is currently no specific treatment for the disease. At present, Huperzine A (HupA) is one of the best drugs used for the treatment of Alzheimer’s disease and has been used in clinical trials for several years in China. HupA was first separated from *Huperzia serrata*, a traditional medicinal herb that is used to cure fever, contusions, strains, hematuria, schizophrenia, and snakebite for several hundreds of years in China, and has been confirmed to have acetylcholinesterase inhibitory activity. With the very slow growth of *H. serrata*, resources are becoming too scarce to meet the need for clinical treatment. Some endophytic fungal strains that produce HupA were isolated from *H. serrate* in previous studies. In this article, the diversity of the endophytic fungal community within *H. serrata* was observed and the relevance to the production of HupA by the host plant was further analyzed. A total of 1167 strains were obtained from the leaves of *H. serrata* followed by the stems (1045) and roots (824). The richness as well as diversity of endophytic fungi within the leaf and stem were higher than in the root. The endophytic fungal community was similar within stems as well as in leaves at all taxonomic levels. The 11 genera (*Derxomyces*, *Lophiostoma*, *Cyphellophora*, *Devriesia*, *Serendipita*, *Kurtzmanomyces*, *Mycosphaerella*, *Conoideocrella*, *Brevicellicium*, *Piskurozyma*, and *Trichomerium*) were positively correlated with HupA content. The correlation index of *Derxomyces* with HupA contents displayed the highest value (CI = 0.92), whereas *Trichomerium* showed the lowest value (CI = 0.02). Through electrospray ionization mass spectrometry (ESI-MS), it was confirmed that the HS7-1 strain could produce HupA and the total alkaloid concentration was 3.7 ug/g. This study will enable us to screen and isolate the strain that can produce HupA and to figure out the correlation between endophytic fungal diversity with HupA content in different plant organs. This can provide new insights into the screening of strains that can produce HupA more effectively.

## 1. Introduction

HuperzineA (HupA), chemical name ((1*R*,9*R*,13*E*)-1-amino-13-ethylidene-11-methyl-6-azatricyclo [7.3.1.02,7] trideca-2(7),3,10-trien-5-one), can be isolated from a traditional Chinese medicinal (TCM) plant *Huperzia serrata* [1,2]. *H. serrata* belongs to *Huperzia* genus, in the Huperziaceae family, which comprises two genera and 150–450 species [3,4], distributed in Eastern, Southern, and Southeast Asia, Oceania, and Central America [5,6]. The dried whole grass has been extensively used as a traditional Chinese medicinal (TCM) herb, named *Qian Ceng Ta*, to cure fever, contusions, strains, hematuria, schizophrenia, and snakebite for several hundreds of years in China [7,8,9,10,11,12]. Modern pharmacological studies have shown that the described medicinal properties of *Qian Ceng Ta* are mainly due to the anticholinesterase activity and anti-inflammatory activity of *Lycopodium*-like alkaloids in *H. serrata* [13,14,15]. Therefore, *H. serrata* has become a standout natural resource to explore new drugs to treat cardiovascular diseases and neuromuscular system disease. In 1986, a new pyridine skeleton lycodine was first isolated from *H. serrata* and named HupA [16]. Subsequently, HupA was found to have anticholinesterase activity and good potential for the treatment of neurodegenerative diseases [16,17,18]. In the USA, HupA, is currently used as a supplement for preventing further memory degeneration [19]. Alzheimer’s disease is a major and snowballing threat to public health, especially to people aged over 65 years old, and has caused serious family and society problems. At present, HupA is one of the best drugs used in the treatment of Alzheimer’s disease (AD) and has been used in clinical trials for several years in China [20,21]. Two randomized control studies have integrated HupA in the formulae and both have reported a significant increase in the Mini-Mental State Examination (MMSE) at the end of the treatment for combined therapy. None of them reported adverse effects and both studies have been included in an updated systematic review of controlled clinical trials of Chinese herbal interventions for people with mild cognitive impairment (MCI) [22]. Until recently [23], the beneficial effects of HupA on AD have been considered to be inconclusive due to poor methodological quality and a small sample size in the preclinical studies and clinical trials. A systematic review on the beneficial and harmful effect of HupA as a treatment of AD [23] found that of 20 randomized control trials (RCTs) included in the qualitative and quantitative synthesis, four meta-analyses and most individual trials favored HupA in scores of cognitive functions. The mean global clinical assessment measured by the Clinical Dementia Rating Scale at 16 weeks in the intervention groups was 0.9 lower. In 12 trials, only mild adverse effects were reported and none reported severe adverse events. Thus, HupA demonstrated a potential beneficial effect for AD compared with the placebo, which is in accordance with previous published reviews on (i) HupA for AD in the Cochrane Register in 2008 [24] and (ii) Chinese herbs (*Gingko biloba* and *Huperzia serrata*) with effects on the development and progression of AD in 2011 [25].

Along with emerging research on plant–microbe interactions, accumulating pieces of evidence suggest that endophytic fungi play important roles in plant growth [26,27]. Previous studies have confirmed that *H. serrata* is rich in endophytic fungi and thus is a natural resource for the screening of a HupA-producer. Endophytes are considered as a kind of microorganism that live inside cells or intercellular spaces of a plant during a certain stage or whole life cycle without showing symptoms of disease [28]. Due to their long-term and complex interaction with host plants, endophytes can produce the same or similar metabolic substances that are produced by host plants [29]. Fungal endophytes producing taxol were isolated from the phloem of *Taxus brevifolia* [30]. This gave new insights into exploring natural HupA from endophytes, and some endophytes that produce HupA were successfully isolated from *H. serrate* [31,32,33,34,35,36]. Endophytic fungi play an important role in the growth, development, fitness benefits, and ecological adaptation of host plants through the assistance, involvement, and regulation of secondary metabolism [37]. Plants with endophytic fungal interactions in natural ecosystems can be mutualistic, symbiotic, and parasitic. Endophytic fungi are transmitted both vertically (systemic) and horizontally (nonsystemic). Vertically transmitted endophytic fungi are mutualistic, while horizontally transmitted endophytes represent antagonism to the host [38]. Environmental, physiological, and genetic control interactions between the endophyte and the plant host are mutualistic [39]. The host’s genetic makeup determines the interaction between fungi and the host [40]. The type of plant–fungal interaction is thus determined by the differential expression of fungal genes in response to the host of the plant, or vice versa. Therefore, depending on slight genetic variations in the genomes of both partners, symbiosis can be positive, negative, or neutral. Nutrient exchange imbalance [39], environmental changes [41], and physiological stress also affect the interaction between fungi and plant. It is suggested that in ecological interactions, secondary metabolites from endophytic fungi play an important role [42]. Plants in which the roots are colonized by endophytes synthesize different plant growth-promoting compounds and phytohormones and thus grow much faster than non-infected plants [43]. The accumulation of secondary metabolites secreted in vivo is insufficient for the expression of disease, but leads to its survival in the ecosystem [44]. For assistance, fungal endophyte produced alkaloids in grasslands are plant defenses; in return, the endophyte receives nutrients and protection within the plant tissues [45]. Endophytic fungi exhibit plant growth-promoting activities and stress resistance as well as the ability to solubilize large amounts of phosphorous (P) from inorganic sources including Ca_3_(PO_4_)_2_ and rock phosphate [46].

Although HupA was originally isolated from *H. serrata*, the content of the compound in the dried herb is less than 0.025% [47,48,49,50]. With the increasing demand for HupA in clinical treatment, *H. serrata* was overexploited. As the plants have a long-life cycle and a small yield can be obtained, scientists have had to seek new resources of HupA. However, as per our knowledge is concerned, the endophytic community within *H. serrata* is lacking in systematic research, and the effects of the community on the growth and development of host plants have been unexplored. At present, there is no systematic study on endophytic communities within different organs of *H. serrate*, the role of endophytic fungi in the biosynthesis of HupA, and the screening and isolation of entophytic fungi that produce HupA. Therefore, it is necessary to study the diversity and composition of endophytes in different tissues within *H. serrata* and the strain that produces a higher concentration of HupA. The Illumina-based high-throughput sequencing technology can comprehensively reveal the diversity and composition of plant-associated endophytes [51]. However, few studies on the diversity and composition of endophytes in *H. serrata* based on high-throughput sequencing have been conducted.

Thus, in this study, Illumina-based high-throughput sequencing analysis of the internal transcribed spacer (ITS) region was conducted to examine the endophytic community in the leaves, stems, and roots of *H. serrata* and the effects of endophytes on the growth, development, and adaption to environments were analyzed, especially in terms of the role of endophytes in the biosynthesis of HupA. Furthermore, we successfully screened and isolated endophytic fungi producing HupA. This will contribute to revealing how the endophytic fungi are involved in the metabolism of HupA and facilitates the efficient isolation of fungal endophytic strains that produce HupA.

## 2. Results

### 2.1. Taxonomic Distribution of Endophytic Fungi

**Figure 1:**
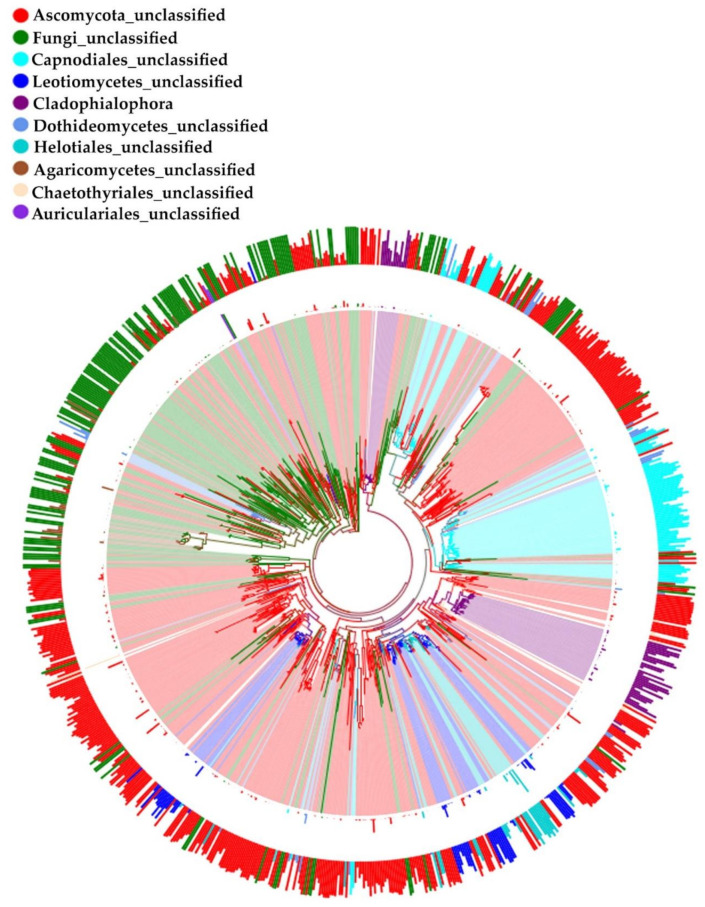
The phylogenetic tree and annotations of species that belong to the 10 genera with the highest abundance. From inside to outside: the first ring displays the neighbor-joining phylogenetic tree based on the internal transcribed spacer (ITS) rDNA alignment of all the OTUs belonging to the 10 operational taxonomic units (OTUs) ranked in different colors. Clade credibility values are indicated at internodes (bootstrap values 1000 replications). Each color represents a genus. The second ring is a representation of the relative abundance values; the value was converted according to the minimum and visualized. The third ring shows the reliability of the species identification, and the height of the columns shows the reliability.

All the endophytic fungal strains isolated from *H. serrata* were cultured on a potato dextrose agar (PDA) medium. A total number of 1800 operational taxonomic units (OTUs) were detected and the DNA of the samples was extracted. The ITS region was amplified using the universal primers ITS1/ITS4. According to the ITS sequence analysis, all the strains were identified and assigned to Ascomycota (73.20%), Basidiomycota (20.61%), Chytridiomycota (0.02%), Rozellomycota (0.02%), Zygomycota (1.41%), and unknown taxa (4.74%). Overall, 1800 OTUs were classified into 278 genera including the unknown. Most of these OTUs were classified as *Cladophialophora* (2.44%), and only the other six genera (*Devriesia*, *Mortierella*, *Dactylonectria*, *Exophiala*, *Serendipita,* and *Pezoloma*) showed frequencies higher than 1%. The identities classified in the top 10 genera were selected and the phylogenetic tree was constructed with the ITS sequences using the neighbor-joining method with bootstrap values of 1000 replications (Figure 1).

### 2.2. Diversity of Fungal Endophytes within Plant Organs

**Figure 2:**
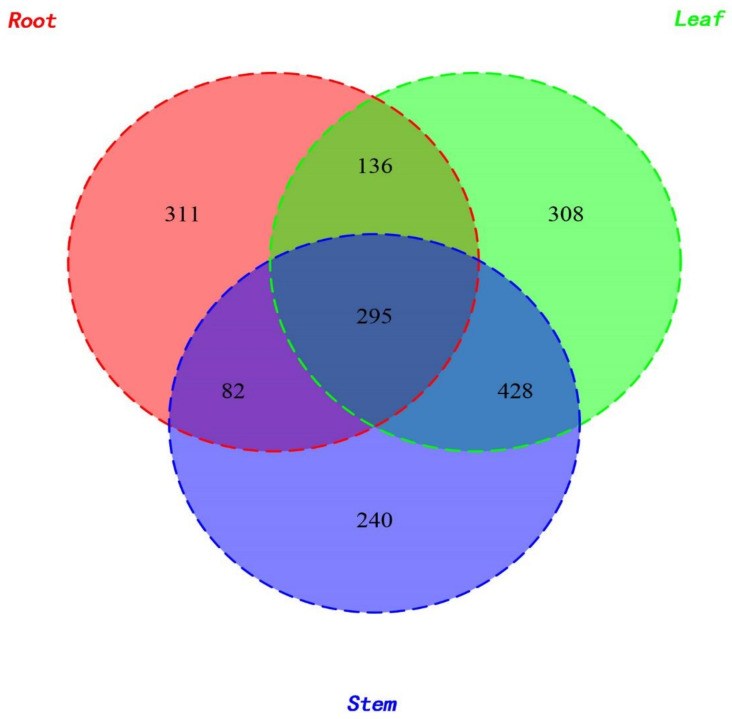
Venn diagram showing the intersections of the identities of the endophytic fungal communities along with the plant organs.

**Figure 3:**
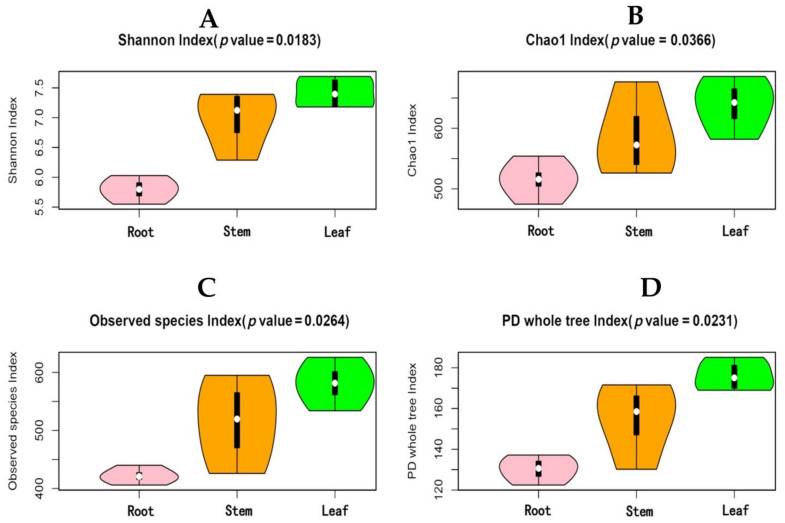
The alpha diversity index of the endophytic fungi isolated from different plant organs of *H. serrata.* (**A**–**D**) are indicating Shannon index, Chao1 index, Observed species index and Phylogenetic diversity (PD) tree index respectively.

**Figure 4:**
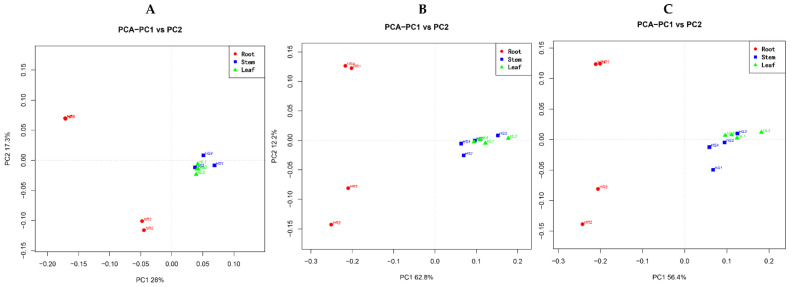
The principal component analysis (PCA) of the community of endophytic fungi within different plant organs including the roots, stems, and leaves. (**A**) PCA was conducted at OUT level, (**B**) PCA was conducted at the genus level, (**C**) PCA was conducted at the family level.

The highest numbers of strains (1167) were obtained from leaves of *H. serrata* followed by the stems (1045) and roots (824). However, the number of the specific strains for a particular plant organ was 311, 308, and 240 for the roots, leaves, and stems, respectively. Only 295 strains were found common in all three organs (Figure 2). Both the diversity of endophytic communities and the alpha diversity values were calculated per plant organ (Figure 3). Overall, the leaves exhibited higher values than roots in all diversity indices employed (Chao1 Index, Shannon Index, Amount of Observed Species (Sobs), and Phylogenetic Diversity (PD) of the whole tree index). The results indicate that the richness as well as diversity of endophytic fungi within the leaf and stem were higher than in the root. The communities of the endophytic fungi within different organs were further analyzed at the family, genus, and species level through principal component analysis (PCA) (Figure 4). The communities of endophytic fungi within the stems were similar to the communities within leaves at all taxonomic levels.

### 2.3. Correlation between Fungal Endophytes and HupA

**Figure 5:**
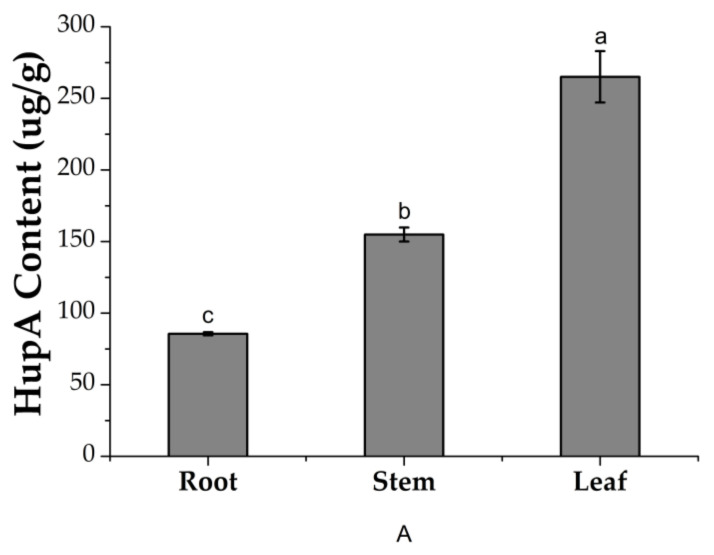
HupA content in different parts including the root, stem, and leaf of *H. serrata*. Whereas, “a, b and c” are representing the level of significance (*p* < 5%).

**Figure 6:**
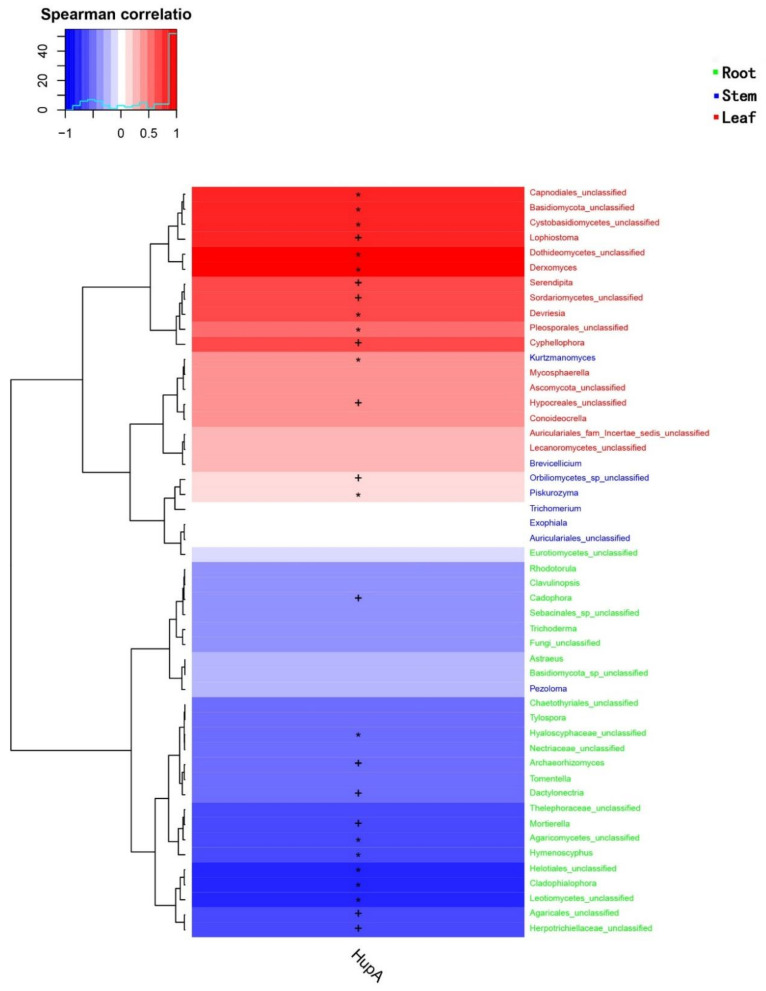
The correlation of HupA content with endophytic fungi within *H. serrata.* Whereas, + indicate *p* < 0.05 and * indicate *p* < 0.01.

The content of HupA in different plant organs was quantified by high performance liquid chromatography (HPLC) (Figure 5). The content of HupA in leaves was 265.01 µg/g, which was significantly higher than in the stems (154.88 µg/g) and roots (85.58 µg/g). The correlation of the contents of HupA with endophytic fungal communities and species was further analyzed and the Spearman correlation index was calculated using the permutational multivariate analysis of variance (PERMANOVA) method. The correlation was demonstrated in Figure 6. There were 11 genera (*Derxomyces*, *Lophiostoma*, *Cyphellophora*, *Devriesia*, *Serendipita*, *Kurtzmanomyces*, *Mycosphaerella*, *Conoideocrella*, *Brevicellicium*, *Piskurozyma*, *Trichomerium*) positively correlated with HupA content. The correlation index of *Derxomyces* with HupA content displayed the highest value (CI = 0.92) whereas *Trichomerium* showed the lowest value (CI = 0.02).

### 2.4. Isolation and Identification of Endophytic Fungi

**Figure 7:**
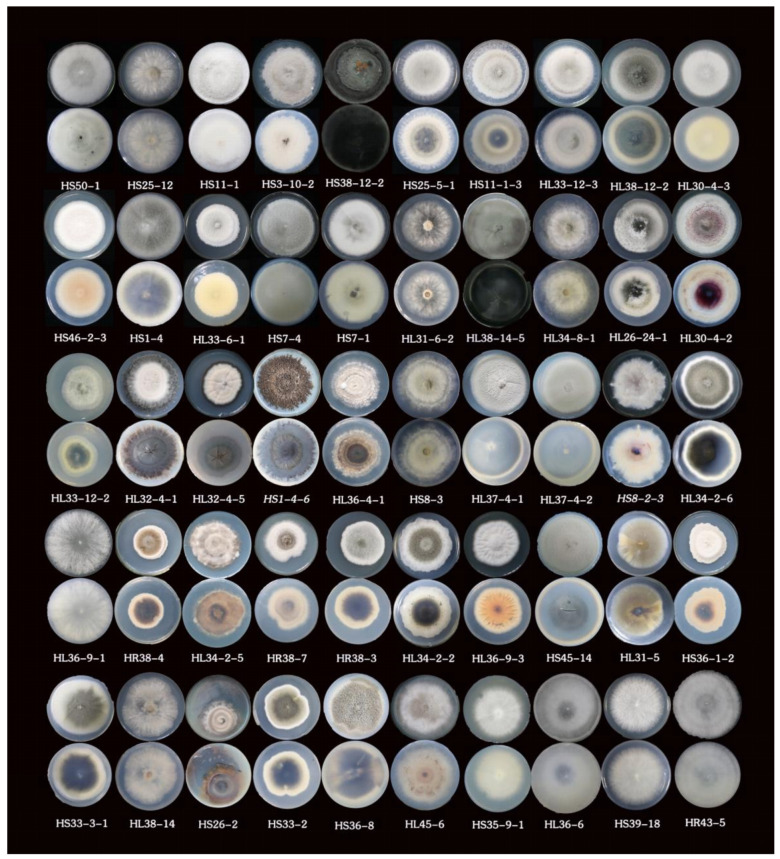
Colonies of the endophytic fungi from H. serrate. HS50-1, HS25-12, HS11-1, HL38-12-2, HS3-10-2, HS38-12-2, HS25-5-1, HS11-1-3, and HL33-12-3 represent *Colletotrichum gloeosporioides*. HL30-4-3, HS46-2-3, HS1-4, HL33-6-1, HS7-4, HS7-1, HL31-6-2, and HL34-8-1 represent *Colletotrichum boninense*. HL26-24-1 represents *Colletotrichum tabaci*, HL30-4-2 shows *Colletotrichum acutata*, HL33-12-2 shows *Fusarium lateritium*, HL32-4-1 and HL32-4-5 represent *Pseudocercospora rubi*, HS1-4-6 shows *Guignardia mangiferae*, HL36-4-1 and HS8-3 show *Leptostroma sp.*, HL37-4-2 and HL37-4-1 show *Arthopyreniaceae sp.*, HS8-2-3 shows *Microsphaeropsis arundinis*, HL34-2-6 and HL36-9-1, HR38-4, HL34-2-5, HR38-7, HR38-3 and HL34-2-2 to *Alternaria alternate*, HL36-9-3 to *Epicoccum nigrum*, HS45-14, HL31-5, HS36-1-2, HS33-3-1, HL38-14, HS26-2, HS33-2, HL45-6 and HS36-8 to *Phoma herbarum*, HL36-6 and HS35-9-1 to *Bjerkandera adustus*, and HS39-18 and HR43-5 to *Trametes versicolor.*

Traditional morphological observation combined with molecular biology is usually used to identify endophytic fungi. For uncommon, non-sporogenous, and non-characteristic fungal colonies, the method of molecular biological identification is usually adopted. If the strains are not distinguished by molecular biological methods, then there are other methods such as morphological observation to identify the strains. A total of 180 single colonies were isolated from the leaves, stems, and roots of *H. serrate*. The morphological and molecular identification of endophytic fungi were accomplished by observing the morphology of each colony, the color of mycelia, whether sporogenesis occurred or not, and by extracting hyphal DNA and PCR amplification for ITS sequencing. Figure 7 shows 50 endophytic fungi of four classes, seven orders, 11 families, and 12 genera. The dominant species were identified as *Colletotrichum boninens*, *Colletotrichum gloeosporioides*, *Alternaria alternata*, and *phoma herbarum. Colletotrichum boninens* and *Colletotrichum gloeosporioides* belong to Sordariomycetes, Glomerellales, Glomerellaceae, and *Colletotrichum*. *Alternaria alternata* belongs to Dothideomycetes, Pleosporales, Pleosporaceae, and *Alternaria,* and *Phomopsis* sp. belongs to Dothideomycetes, Pleosporales, Didymellaceae, and *Phoma*. Phylogenetic tree based on ITS sequences of 50 endophytic fungi in *H. serrate* is shown in Figure S1. Identity of the endophytes from *H. serrata* by the ITS sequences analysis is shown in Table S1.

### 2.5. Screening of Endophytic Fungi Producing HupA

**Figure 8:**
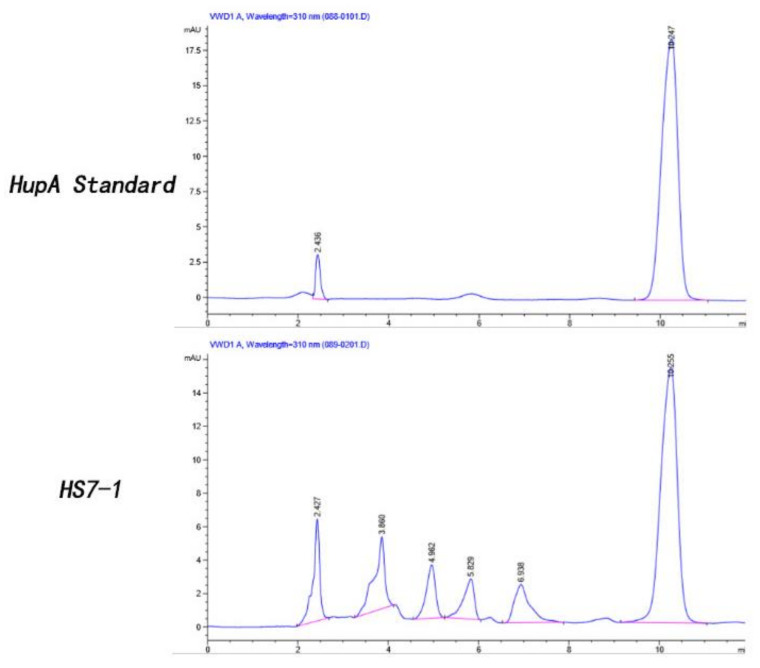
HPLC histogram of the standard HupA and HS7-1 strain crude alkaloid extract.

HupA is a secondary metabolite [52] that is biosynthesized during the fermentation of endophytic fungus [53]. Therefore, it is necessary to use sensitive and reliable high-performance liquid-phase extraction for qualitative detection. The standard gradient curve of HupA shows that the method is reliable and sensitive for the qualitative and quantitative determination of HupA (Figure S2). The 180 strains of isolated endophytes were cultured in shaking flasks. The total alkaloid obtained from the mycelia was extracted. The HupA standard was used as a control. HPLC detection was performed to verify whether HupA was produced. Fortunately, HS7-1 strain was checked to produce HupA with the replication of three generations. HupA content of the fourth generation was further measured by HPLC. In Figure 8, the characteristic absorption peak at 310 nm of the mycelial alkaloid extracted from a strain *Colletotrichum boninense* HS7-1 was determined by HPLC. This proved that the HS7-1 strain could produce HupA.

From the data in Table 1, it was observed that the yield of HupA per gram of dried mycelia was 3.7 μg/g, and for the control, the detected concentration was 4.4 μg/g.

### 2.6. Detection of Metabolites of the HS7-1 Strain by Electrospray Ionization Mass Spectrometry (ESI-MS)

**Figure 9:**
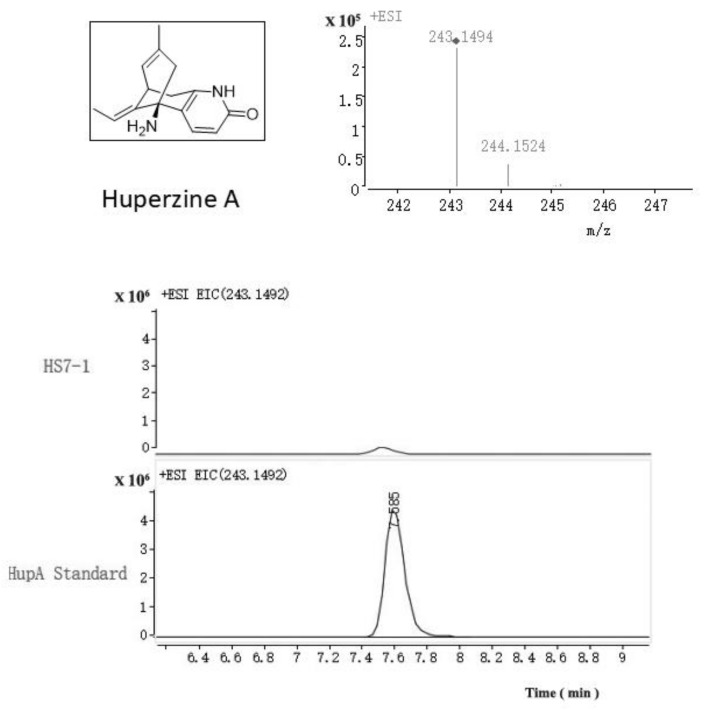
Detection of the fungal HupA of the HS7-1 strain by Electrospray Ionization Mass Spectrometry (ESI-MS) analysis.

The results of the electrospray ionization mass spectrometry (ESI-MS) (Figure 9) showed that HS7-1 was consistent with the standard mass spectrum. The results showed that HS7-1 could produce HupA with the same molecular weight and the same molecular structure. However, after repeated experiments, it was proven that the HS7-1 (*Colletotrichum boninense*) strain had degradation, low mycelial growth, low content of HupA, and poor repeatability. To obtain a high yield of HupA, the fermentation conditions of the HS7-1 strain were optimized to increase the content of HupA.

## 3. Discussion

*H. serrata* has been widely cultivated in China. It is in danger of extinction due to its medicinal values and the economic importance of its bioactive ingredients such as HupA. Much effort has been put into cultivation and tissue culture for the production of *H. serrata* to solve the problem, but these strategies are generally ineffective. The reason is that *H. serrata* actually contains very low HupA contents (*c.* 0.007%), has a very short distribution, and grows quite slowly. The tissue harvested for medicinal use takes at least 15 years from spore germination through the gametophyte stage to eventually reach the mature sporophyte stage. Although other Huperziaceae species are much more difficult to obtain and are even rarer in nature than *H. serrata*, they are much less attractive as natural sources of HupA candidates. Moreover, propagated in vitro tissues could yield much higher amounts of HupA than the natural plant, but it would not be feasible to satisfy the needs of the pharmaceutical industry [36]. Therefore, there should be a new approach to enhance the production of HupA within different parts of *H. serrata.* This could comprehensively explain the mechanism as well as which plant part has great potential for the biosynthesis of HupA. Researchers are trying to explore the endophytic fungi within *H. serrata*. Endophytic fungi develop inside their hosts despite causing obvious signs of disease [54,55], and growth in this habitat requires constant metabolic interaction between both the fungus and the host. Moreover, secondary metabolites synthesized by a fungus may conform to their respective ecological niche, for example, plant-pathogen mycotoxins [56]. It is necessary to use classical taxonomy and molecular methods to examine and classify endophytic members of this diverse community of fungi. From our knowledge, the literature review shows studies where more than 100 to 400 species were isolated and screened with several strains producing HupA. By use of molecular approaches alone such as ITS region sequence analysis, this can be hindered by the inclusion of unclear sequences that have been deposited in genomic databases. The rDNA ITS region, however, is known as the best region for fungal species identification and has been designated as a fungal barcode [57]. In contrast, the emergence of a wide number of endophytic fungi hampers recognition using morphological taxonomy alone. In history, many of the isolated endophytic fungi do not sporulate and are thus designated as sterilia of mycelia.

In this study, we isolated 1800 operational taxonomic units (OTUs) belonging to 278 genera, and the endophytic fungal communities within different plant organs such as the roots, stems, and leaves of *H. serrata* were analyzed. It has been suggested that metabolites produced by a fungus may differ with the biotope in which it grows and to which it is adapted. The reason is that endophytic fungi contain many types of exoenzymes, which are required to colonize the hosts and grow well in the host’s apo-plastic washing fluid. Moreover, it is supposed that interaction between fungal endophytes with the host plant is categorized by a tuned equilibrium of plant defense and fungal virulence. The exoenzymes and endophytic fungal–host equilibrium are varied in different biotopes [58]. 

In the present study, higher values of diversity indices of endophytic fungi were found for leaves compared to roots. The communities of endophytic fungi within leaves were similar to the communities within stems at all taxonomic levels. Leaves yielded the highest amount of HupA, followed by stems and roots. Luo et al. (2010) reported that genes involved in lycopodium alkaloid biosynthesis were expressed more abundantly in the leaves than in roots. Thus, we may infer that the endophytic microbiome related to leaves and stems might be involved in the production of HupA, both as some self-producers of the antagonistic alkaloid as well as eliciting the host response [4]. Several researchers have analyzed endophyte distribution patterns in plant tissues and, probably in most cases, foliar endophytes have been investigated [59,60]. The composition of the endophytic assemblage species and the frequency of infection differed by host species, site characteristics such as altitude, exposure, related vegetation type of tissue [61], and phase of tissue [62]. Usually, a relatively consistent group of fungal genera and species, distinguished by a few dominant species, contains the assemblage of foliar endophytes for a given host. Current research also supports this assumption [62]. Therefore, it is important to keep in mind that leaf tissues are favored by endophytic fungi, although the reason is still unknown. A large level of tissue specificity for endophytic fungi has been reported previously [63,64]. To show the diversity and richness of the fungal community within samples, Chao1, Shannon, Amount of Observed Species (Sobs), and Phylogenetic Diversity (PD) indices were used. The Chao1 and Shannon indexes display the species richness; a higher index reflects the fungal community richness. The Sobs and PD indexes indicate the fungal diversity within the plant parts. It has been revealed from the results that species richness and diversity of fungal endophytes within leaves and stems presented higher values than in the roots. This is also a reasonable agreement to other previous studies of different plants that contain higher species richness as well as diversity in the leaves and stems than in the roots [65,66,67]. While the colonization of the above-ground organs by non-clavicipitaceous endophytes is considered primarily intercellular and local [68], roots endophytes are typically widespread and systemic and can be intracellular or intercellular (e.g., endomycorrhizal and ectomycorrhizal fungi). If endophytic fungi colonize the shoot intercellularly, they may be able to use the apoplastic fluid as a supply of nutrients [69]. The endophytic fungi could mediate the growth, development, and resistance of the host plant [38]. Aside from *H. serrate*-fungal synergy, *H. serrate*–bacterial interaction is also an important source of metabolite biosynthesis. In a study, it was observed that *H. serrate* contains a rich amount of bacterial diversity, particularly in the above ground plant parts. Eleven potential novel species belonged to the genera *Amycolatopsis*, *Luteimicrobium*, *Glaciihabitans*, *Angustibacter*, *Massilia*, *Arthrobacter*, *Curtobacterium*, *Frondihabitns*, *Jatrophihabitans*, *Naumannella*, and *Tardiphaga*, and one novel genus of the *Dermacoccaceae* family were dominant in *H. serrate* [70]. It is suggested that some fungal strains show antimicrobial activity against bacteria such as *Bacillus cereus* (Gram-positive) and *E. coli* (Gram-negative) and effectively decreased nematode infections in certain plant roots [71,72]. Endophytic fungi live in plants for a certain stage in their life cycle, but do not cause visible symptoms in plant tissues. Studies suggest that endophytic fungi and the host plant have a similar pathway for secondary metabolite synthesis and may generate the same or similar physiologically bioactive compounds [73]. The latest reports have shown that the HupA compound is produced by many types of endophytic fungi, hence providing a potential alternative option as fungi are far more conducive than plants due to their simpler genetics and ease of manipulation [74]. This technique is expected to reduce the burden on wild *H. serrata*, thus allowing the efficient use of HupA as a medicinal resource. HupA is an extract of *H. serrate*. HupA was mainly produced and restored within the *H. serrata* leaves and stimulated the growth and resistance of the plant host [75]. Our report revealed that the content of HupA was different in all three organs of *H. serrata*. The highest amount was found in the leaves (265.01 μg/g) while the roots had slightly smaller quantities (85.58 μg/g). The stems contained intermediate levels (154.88 μg/g). This is also a positive correlation with other published studies as the leaves contained higher HupA content [76]. For HupA isolation, the best tissues are the leaves, followed by the stems. This means that it is not appropriate to harvest the whole plant, but that it is possible to make cuttings instead, possibly enabling the root system to regenerate new aerial parts of the plant. If this approach is to be adopted by those who cultivate *H. serrata* for medicinal use, and if the collection were to continue, the wild populations of these plants could be avoided. However, it is very unlikely that the strategy of taking only cuttings during harvesting, especially by poachers, will be adopted because of the ease with which the whole plant is harvested and traditional medicine practices, where the whole plant is used as medicine. However, preparations made entirely from *H. serrata* leaves will possibly be more suitable for use as a dietary replacement for the treatment of Alzheimer’s disease symptoms. HupA-producing strains have been isolated from fungi living in *H. serrata* in recent years and HupA production has been ascertained by Ju et al. (2009), who optimized certain growth conditions and isolated endophytic fungal strains producing HupA such as *Blastomyces* sp. HA15 and *Botrytis* sp. HA23 from *Phlegmariurus Crytomerianus*. The total quantity of HupA was recorded as 20 to 30 µg/g of dry mycelial [77]. In another study, a HupA-generating endophytic fungal strain SLF14 was isolated with the production of 142.6 μg/g of dry mycelia from *H. serrata* by Zhu et al. (2010) [36]. Existing experiments have shown that endophytic fungi developing HupA can be isolated from the Hupziaceae species. It was observed that the yield of HupA per gram of (*Colletotrichum boninense*) strain HS7-1 dried mycelia was 3.7 μg/g. This provides new outlets for the therapeutic use of HupA. However, this amount of production is also far from industrial production. To obtain a high yield of HupA, the fermentation conditions of the HS7-1 strain must be optimized to increase the content of HupA.

Shu et al. (2014) isolated an endophytic fungus strain that produced HupA from *H. serrata*, and the strain was identified as *Colletotrichum gloeosporioides* [32]. In this research, 11 genera were positively correlated to HupA production. *Colletotrichum* was also positively correlated to HupA production. Our laboratory isolated the HupA-producing strains of *Colletotrichum* sp. and calculated HupA production. Our research provides insights for the isolation and screening of endophytic fungi that produce HupA. The main objective was an improvement by using selective breeding methods for high-yielding strains at the genetic or metabolic stage based on *Colletotrichum* sp. The fermentation conditions of the HS7-1 strain should be optimized to increase the content of HupA. Therefore, how to improve the strains to highly express HupA within the host is the trend in this area.

## 4. Materials and Methods

### 4.1. Collection and Surface Sterilization of H. serrata Plants

*H. serrata* was collected from Shizhu County (N108.4585°, E30.3137°, altitude 1475 m), Chongqing City, China and deposited in the Molecular Pharmacognosy Lab, Huazhong Agricultural University. The collected plants were identified by Prof. Keqin Wang, a pharmacognosist at the Academy of Chinese Traditional Medicine in Hubei Province, China. The whole healthy *H. serrata* was divided into the leaves, stems, and roots after being cleaned of debris with tap water. The process of surface sterilization was used to eliminate epiphytic microorganisms from the plant. Samples were immersed in 75% ethanol, sterilized for 5 min, transferred to 1.5% HgCl_2_, and then sterilized for 1.5 min, followed by rinsing with distilled water five times. The last rinsing water was collected and used as a control. All samples were separated into two categories. The first category was used for the quantification of HupA contents, and the other one was used for endophytic fungal isolation.

### 4.2. Endophytic Fungal Isolation

The surface-sterilized roots and stems were cut into 10 mm and the leaves into 5 mm × 5 mm parts under sterilized conditions. The plant’s parts were then placed on a potato dextrose agar (PDA) medium containing 200 mg·L^−1^ ampicillin and streptomycin to avoid bacterial contamination and incubated at 25 °C for 7–10 days. As the fungal outgrowth of the plant tissues appeared, morphological characteristics of the fungi were observed. Only fungi with distinct morphological features were subcultured. For further screening and identification, the strains were preserved in 15% glycerol at −80 °C.

### 4.3. Identification of Fungal Endophytes

A preliminary classification was made before taxonomic identification to avoid the selection of identical strains resulting from the same plant, distinguishing isolates into morpho-types. A strain was inoculated on a PDA Petri plate for the microscopic observations and a sterilized cover slide was set at two centimeters. The slide was removed after the growth of the fungi partially covered the cover slide, inverted on a cotton blue slide (for the light-colored colonies), and examined under the microscope.

A Nikon Eclipse 55i biological microscope was used to digitally capture photomicrographs. Molecular identification was carried out in the following steps. First, the mycelia were harvested, ground in liquid nitrogen, and the total genomic DNA was extracted using the Cetyltrimethylammonium Bromide (CTAB) method with certain modifications [78]. Using universal primers ITS1 (5′-TCCGTAGGTGAACCTGCGG-3′) and ITS4 (5′-TCCTCCGCTTATTGATATGC-3′), the ITS region was amplified. PCRs were carried out in a final volume of 25 µL containing 100 ng genomic DNA, 50 mM KCl, 10 mM Tris–HCl, 0.2 mM dNTP, 1.5 mM MgCl_2_, and 1 U Taq DNA Polymerase (Promega, Madison, WI, USA). The PCRs conditions were as follows: 94 °C for 3 min; 30 cycles of 94 °C for 30 s, 58 °C for 30 s, and 72 °C for 1 min; and a final extension at 72 °C for 10 min [32]. The 1.6 percent agarose gel electrophoresis was used to check the PCR products and then cloned using the pGEM^®^-T Easy Vector System (Promega, Madison, WI, USA), and transformed into *Escherichia coli* colony DH5a (Invitrogen Life Technologies; Carlsbad, CA, USA). The plasmid carrying the fragment of interest was purified from *E. coli* and sent for sequencing to BGI (Shenzhen, China). The sequences were run through the BLASTN search page using the Megablast program (National Center for Biotechnology Information; Bethesda MD, USA) where the most alike hits and their accession numbers were gained. MEGA version 7.0 software (Pennsylvania State University: USA, 1993) was used to perform phylogenetic analysis. ClustalW was used to conduct multiple alignments. The evolutionary history was inferred using the neighbor-jointing approach of 1000 bootstrap replications, and to distinguish the most similar relationship, Kimura’s two-parameter models was used.

### 4.4. Quantification of HupA in H. serrata

The *H. serrata* plant samples were dried at 80 °C for 24 h, pulverized, and precisely weighted in 2 g. The powder was dissolved in 40 mL pH = 3 HCl and stirred for 10 h at room temperature, then sonicated for 1 h at 50 °C. To the filtered solution, 20 mL HCl, pH 3 were added and extracted. All the extracts were collected with a pH of 9 adjusted with NH_4_OH. Then, the same volume of chloroform was added to the extracts and extracted twice. Using the rotary evaporator, the chloroform layers were combined and evaporated into dryness at low pressure. The collected residues were reconstituted into 10 mL of methanol after evaporation and purified through a filter syringe (0.45 μm) to perform HPLC. Each sample was extracted and quantified with three replications. HupA content was determined using an Agilent HPLC 1260 series (Agilent, Santa Clara, CA, USA) with a Wondasil C18 column (4.6 mm × 250 mm, 5um; Shimadzu, Tokyo, Japan). The column temperature was adjusted at 25 °C. The flow rate was 1 mL/min and using 80 mM ammonium acetate (pH 6.0)–methanol (65:35, *v*/*v*) as the mobile phase. The samples were detected at 310 nm. The HupA contents in the samples were quantified employing the standard curve created from the HupA standard (purchased from Shanghai Tauto Biotechnology Co. Ltd., Shanghai, China) in a concentration range of 5.87–29.33 µg/mL.

### 4.5. Endophytic Fungal Diversity Analysis

The relative proportion was determined as a percentage of the number of isolates belonging to a species or OTU divided by the total isolates recovered from all samples. Species richness (S) among fungal endophytes was calculated for each type of plant organ (i.e., leaves, roots, stems). From the following Equation (1), Shannon’s diversity index (H) was calculated:

(1) $$H=-\Sigma \left(\mathrm{pi}\;\times \;\ln\;\mathrm{pi}\right)$$

where pi is indicating the relative proportion of species I in a specific organ.

Principal component analysis (PCA) and correlation were carried out by R statistics software v3.4.0 (Total Genomics Solution, Shenzhen, China, 2014). PCA reveals the interrelationships among fungal endophytes recovered from certain organs and correlation shows the concurrence between fungal diversity and HupA content for a certain fungal community within a plant organ. The Duncan multiple range test (DMRT) was conducted to measure the significant variations between values at the *p* = 0.05 level using SAS v9.4 (Total Genomics Solution, Shenzhen, China, 2014). Microsoft Excel 2013 was used to draw graphs.

### 4.6. Morphological Identification Method

The morphological identification is principally based on morphological characteristics. Taking advantage of the differences in the morphological characteristics of fungi in the appropriate medium (mycelial color, their microscopic characteristics, fruiting body characteristics, colony texture, pigment, etc.) and asexual stage conidia and its morphological characteristics during development, ascomycete type in sexual stage, ascomycete shape, and color characteristics were identified. The endophytic strains stored AT-80 °C were inoculated on a PDA plate and activated in an incubator at 25 °C; the mycelia were taken from the edge of the colony with a 6 mm diameter aseptic perforator. The mycelium was then transplanted onto a new PDA plate. The inoculated plates were cultured in an incubator at 28 °C. The colony morphology, mycelium color, pigment production, spores, and sclerotia were observed day by day.

### 4.7. Fermentation of Endophytic Fungi and Extraction of Huperzine A from Huperzia serrate

The endophytic fungal mycelia were inoculated into a PDB liquid medium for flask culture. HupA is a secondary metabolite, and it is necessary to culture it for 15 days at 28 °C for 150 rpm to collect the mycelial sample producing HupA. The mixture was then filtered through a Brinell funnel, dried at 40 °C, dissolved for 45 min in 0.5% hydrochloric acid at 1:30 by weight/volume ratio, and soaked overnight. After filtration, the supernatant was used to adjust the pH to 9.0 with concentrated ammonia water, then extracted three times with chloroform of equal volume, combined with chloroform solution, and evaporated by rotation until the chloroform evaporated. The crude alkaloid samples were obtained by dissolving the mycelia in 1:1 methanol, and stored in a refrigerator at 4 °C.

### 4.8. Determination of Alkaloids by Electrospray Ionization Mass Spectrometry (ESI-MS)

The alkaloid extracts of endophytes from *H. serrata* were separated and purified by high-speed counter-current chromatography. The molecular weight of the compound was determined and compared with the mass spectrogram of HupA. The ESI-MS conditions were as follows: liquid chromatographic column: Agilent ZORBAX Eclipse XDB-C18 4.6 mm × 150 mm 5-Micron and mobile phase A: 10 mMNH_4_AC (pH 3.5). Aqueous Solution: methanol, gradient elution: T: 0–20–30 min B%: 5%–56%–56%, Column temperature: 30 °C, Detector: DAD (190–400 nm), Detection wavelength: 310 nm, Flow rate: 1.0 mL/min, Ion Source: ESI. Positive ion detection mode; Scanning range (*m*/*z*): 80~300, Ion source temperature (°C): 250, Atomization gas flow rate (psi): 40, Dry gas flow rate (L/min): 10, Capillary voltage (V): 4500, capillary outlet voltage (V): 109, Mass spectrometry data processing LC/MS Trap software, version 5.2 (Total Genomics Solution, Shenzhen, China, 2014). 

## 5. Conclusions

This study showed that the endophytic fungal diversity was higher in the leaves of *H. serrate*. The endophytic fungi of this plant are a very important resource to produce HupA. The relative abundance of the endophytic fungi may have a positive correlation to chemical contents in the plant organs.

## Figures and Tables

**Table 1:** HPLC statistics of the standard HupA and HS7-1 strain crude alkaloid extract.

Samples	Retention Time (Min)	Concentration (μg/g)	Area (mAu·sec)
Control	10.247	4.4	459.66324
HS7-1	10.255	3.7	388.57199

Alkaloid quantity along with retention time and the peak area of HPLC analysis. HS7-1 represents *Colletotrichum boninense*.

## Data Availability

Not applicable.

## Supplementary Materials

Figure S1: Phylogenetic tree based on ITS sequences of 50 endophytic fungi in *H. serrata*. Figure S2: Standard HupA linear equation. Table S1: Identity of the endophytes from *H. serrata* by the ITS sequences analysis.
