# Supplementary material for: Endophytic Fungal Community of Huperzia serrata: Diversity and Relevance to the Production of Huperzine A by the Plant Host
Source: Molecules. 2021 Feb 8;26(4):892. doi: 10.3390/molecules26040892 (PMC7914787; doi:10.3390/molecules26040892)
Supplement: Supplementary file 1 [file molecules-26-00892-s001.pdf]

Table 1. Identity of the endophytes from *H. serrata* by the ITS sequences analysis

Isolate (GenBank Acc. No.)	Identity to the closest species (GenBank Acc.No.)
HS50-1 (MK424463)	99% to *Colletotrichum gloeosporioides* (KC010549.1)
HS25-12 (MK424449)	99% to *Colletotrichum gloeosporioides* (KC010549.1)
HS11-1 (MK424446)	100% to *Colletotrichum gloeosporioides* (KC010549.1)
HS3-10-2 (MK424423)	99% to *Colletotrichum gloeosporioides* (KC010549.1)
HS38-12-2 (MK424409)	100% to *Colletotrichum gloeosporioides* (KC010549.1)
HS25-5-1 (MK424406)	100% to *Colletotrichum gloeosporioides* (KC010549.1)
HS11-1-3 (MK424405)	100% to *Colletotrichum gloeosporioides* (KC010549.1)
HL38-12-2 (MK629743)	100% to *Colletotrichum gloeosporioides* (KC010549.1)
HL33-12-3 (MK424400)	99% to *Colletotrichum gloeosporioides* (KC010549.1)
HS46-2-3 (MK424428)	100% to *Colletotrichum boninense* (FJ968604.1)
HS1-4 (MK424418)	100% to *Colletotrichum boninense* (FJ968604.1)
HL33-6-1 (MK424419)	100% to *Colletotrichum boninense* (FJ968604.1)
HS7-4 (MK424424)	100% to *Colletotrichum boninense* (FJ968604.1)
HS7-1 (MK424444)	100% to *Colletotrichum boninense* (FJ968604.1)
HL31-6-2 (MK424450)	100% to *Colletotrichum boninense* (FJ968604.1)
HL34-8-1 (MK424458)	100% to *Colletotrichum boninense* (FJ968604.1)
HL30-4-3 (MK424464)	100% to *Colletotrichum boninense* (FJ968604.1)
HL38-14-5 (MK424460)	100% to *Colletotrichum dematium* (AB455254.1)
HL26-24-1 (MK424411)	100% to *Colletotrichum tabaci* (JQ005763.1)
HL30-4-2 (MK424398)	100% to *Colletotrichum acutata* (KR180397.1)
HL33-12-2 (MK424467)	99% to *Fusarium lateritium* (KX494872.1)
HL32-4-1 (MK424399)	99% to *Pseudocercospora rubi* (MH855672.1)
HL32-4-5 (MK424414)	99% to *Pseudocercospora rubi* (JX436789.1)
HS1-4-6 (MK629744)	100% to *Guignardia mangiferae* (JX436789.1)
HL36-4-1 (MK424402)	100% to *Leptostroma* sp. (KC354586.1)
HS8-3 (MK424404)	100% to *Leptostroma* sp. (KC354586.1)
HL37-4-1 (MK629745)	99% to *Arthopyreniaceae* sp. (JN253508.1)
HL37-4-2 (MK424403)	100% to *Arthopyreniaceae* sp. (JN253508.1)
HS8-2-3 (MK424445)	100% to *Microsphaeropsis arundinis* (KX510124.1)

HL34-2-6 (MK424436)	100% to *Alternaria alternat* (KT345696.1)
HL36-9-1 (MK424439)	100% to *Alternaria alternata* (KT345696.1)
HR38-4 (MK424432)	100% to *Alternaria alternata* (KT345696.1
HL34-2-5 (MK424421)	99% to *Alternaria alternata* (KT345696.1)
HR38-7 (MK424417)	99% to *Alternaria alternata* (KT345696.1)
HR38-3 (MK424416)	99% to *Alternaria alternata* (KT345696.1)
HL34-2-2 (MK424401)	99% to *Alternaria alternata* (KT345696.1)
HL36-9-3 (MK629746)	100% to *Epicoccum nigrum* (MK370650.1)
HS45-14 (MK424410)	100% to *Phoma herbarum* (MK370675.1)
HL31-5 (MK424412)	100% to *Phoma herbarum* (MK370675.1)
HS36-1-2 (MK424408)	99% to *phoma herbarum* (MK370675.1)
HS33-3-1 (MK424426)	99% to *phoma herbarum* (MK370675.1)
HL38-14 (MK424429)	99% to *phoma herbarum* (MK370675.1)
HS26-2 (MK424434)	99% to *phoma herbarum* (MK370675.1)
HS33-2 (MK424454)	99% to *phoma herbarum* (MK370675.1)
HS36-8 (MK424456)	99% to *phoma herbarum* (MK370675.1)
HL45-6 (MK424461)	99% to *phoma herbarum* (MK370675.1)
HS35-9-1 (MK424407)	99% to *Bjerkandera adusta* (MH815138.1)
HL36-6 (MK424438)	100% to *Bjerkandera adusta* (MH815138.11)
HS39-18 (MK424435)	100% to *Trametes versicolor* (KY628332.1)
HR43-5 (MK424443)	100% to *Trametes versicolor* (KY628332.1)

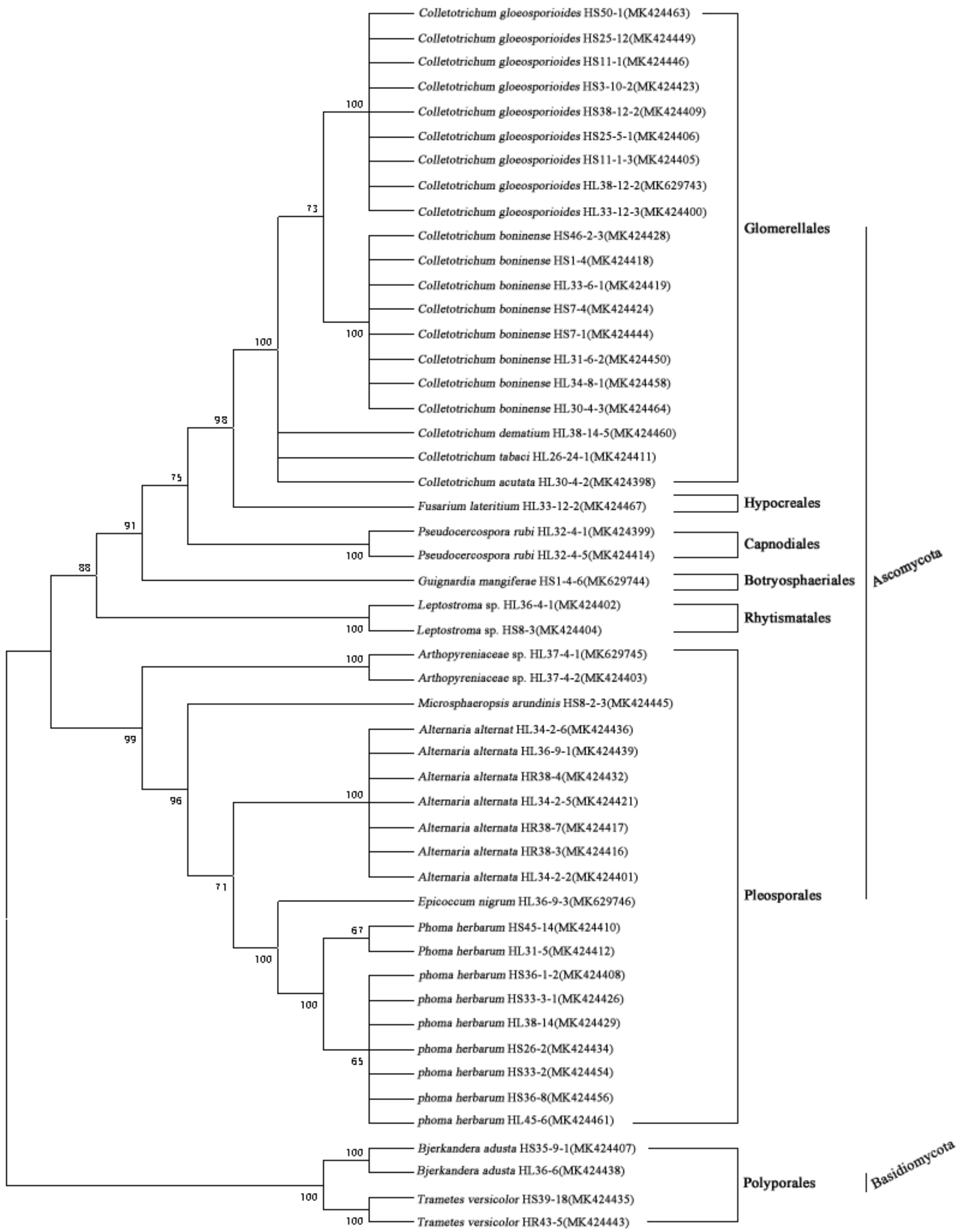

Figure 1. Phylogenetic tree based on ITS sequences of 50 endophytic fungi in *H. serrata*

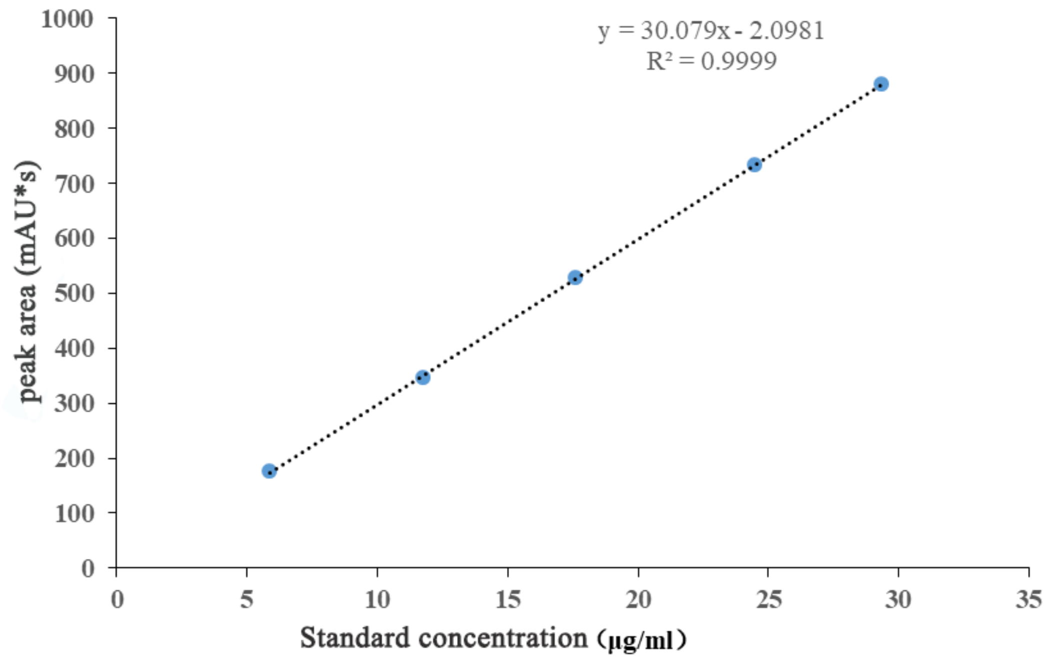

Figure 1. Standard HupA linear equation
